# Exposure to fitness role models on social media and social physique anxiety among college students: the sequential mediating roles of upward body comparison and body self-discrepancy

**DOI:** 10.3389/fpsyg.2026.1924236

**Published:** 2026-09-17

**Authors:** Runyu Wang, Hongfei Li, Yuhong Wang, Chaohu He

**Affiliations:** 1 College of Humanities, Arts and Social Sciences, University of Exeter, Exeter, United Kingdom; 2 School of Physical Education, Kunming University, Kunming, China

**Keywords:** body self-discrepancy, college students, social media fitness role models, social physique anxiety, theoretically ordered indirect associations, upward body comparison

## Abstract

**Introduction:**

Fitness role models on social media combine exercise- and health-oriented communication with highly visible body ideals. This study examined associations among exposure to fitness role models, upward body comparison, body self-discrepancy, and social physique anxiety among college students, including separate and theoretically ordered indirect associations.

**Methods:**

The study comprised a preliminary measurement phase and a formal cross-sectional survey. A pilot study with 162 valid responses examined item performance, internal consistency, and dimensional structure. The formal survey retained 528 valid responses from college students in Yunnan Province, China. Exposure to fitness role models was modeled as a higher-order construct comprising passive browsing and active participation. Confirmatory factor analysis evaluated the measurement model, and a latent-variable structural equation model with covariate paths to social physique anxiety tested the hypothesized associations. Standardized direct and indirect associations were estimated using 5,000 bootstrap resamples. Two unadjusted PROCESS Model 6 sensitivity analyses used observed scores, including an alternative dimension-level discrepancy × importance product score for body self-discrepancy.

**Results:**

Exposure to fitness role models was positively associated with upward body comparison (β = 0.622), body self-discrepancy (β = 0.332), and social physique anxiety (β = 0.263). Upward body comparison was positively associated with body self-discrepancy (β = 0.314) and social physique anxiety (β = 0.131), and body self-discrepancy was positively associated with social physique anxiety (β = 0.285). Bootstrap confidence intervals excluded zero for the standardized indirect associations through upward body comparison (effect = 0.081, 95% CI [0.006, 0.145]), body self-discrepancy (effect = 0.095, 95% CI [0.027, 0.194]), and their theoretically ordered combination (effect = 0.056, 95% CI [0.014, 0.111]). The standardized direct association also remained statistically different from zero (effect = 0.263, 95% CI [0.114, 0.349]). Supplementary observed-score analyses produced broadly similar positive indirect-association patterns.

**Discussion:**

Greater exposure to fitness role models was concurrently associated with stronger upward body comparison, greater body self-discrepancy, and higher social physique anxiety. These findings support integrating externally referenced social comparison and internally referenced body self-discrepancy. Because the study was cross-sectional and self-reported, causal direction and temporal ordering cannot be established.

## 1 Introduction

Social media has become a prominent environment in which young adults encounter information about exercise, health, fitness, and the body. Within this environment, fitness role models—including fitness influencers, exercise content creators, athletic peers, and ordinary users who publicly share training routines, exercise performance, body transformations, diet practices, or fitness lifestyles—may function simultaneously as sources of health-oriented information and as visible standards for body evaluation. Fitness influencers, for example, have been examined as digital health communicators whose perceived credibility and related characteristics are associated with users’ attitudes and exercise intentions (Durau et al., 2024). At the same time, the highly visual nature of social media means that fitness-related communication frequently places bodies, appearance, training outcomes, and lifestyle practices at the center of users’ attention.

Exposure to fitness role models may occur through different patterns of social media use. Research on social media behavior has distinguished passive content consumption from more active forms of engagement (Gerson et al., 2017). In the present context, passive browsing refers to incidental or platform-facilitated encounters with fitness-role-model content during routine social media use, such as exposure through recommendation pages, trending pages, or content feeds. Active participation, by contrast, refers to more intentional engagement, including searching for, following, viewing, saving, or interacting with fitness-related content. These modes differ principally in intentionality and degree of engagement: passive browsing can expose users to fitness standards without deliberate information seeking, whereas active participation involves greater self-selection into fitness-related content environments. Fitness-related social media also provides salient targets for social comparison, as demonstrated in research examining fitness-app users’ comparisons with fitness postings (Kim, 2022). Although passive and active forms of exposure are conceptually distinct, both provide opportunities to encounter fitness-related bodies, lifestyles, and evaluative standards. The present study therefore treats them as first-order dimensions of a broader fitness-role-model exposure construct rather than as interchangeable indicators of general social media use.

Concerns about the body-evaluative implications of such exposure are grounded in a much longer literature on idealized media imagery. A meta-analytic review of experimental research found that exposure to thin-ideal media images was associated with more negative body-image evaluations, particularly among individuals with pre-existing body-image vulnerabilities (Groesz et al., 2002). Similar concerns have emerged in contemporary social media environments. Fitspiration, although commonly framed as encouragement to exercise and adopt healthy habits, often presents relatively narrow appearance ideals involving thinness, visible muscularity, low body fat, or a toned physique. In an experimental study, women exposed to Instagram fitspiration images reported greater body dissatisfaction and negative mood and lower state appearance self-esteem than women exposed to travel images (Tiggemann and Zaccardo, 2015). Systematic reviews have likewise concluded that exposure to fitspiration and other idealized social-media imagery is frequently associated with body dissatisfaction, negative mood, and related body-image concerns (Fioravanti et al., 2022; Jerónimo and Carraça, 2022).

The body ideals represented in fitness media are not necessarily identical across individuals or gendered sociocultural contexts. Much foundational work on media and body image focused on the thin ideal among women, whereas research involving men has highlighted muscularity and lean-muscular appearance. In an experimental study of college men, exposure to advertisements featuring muscular men resulted in a greater discrepancy between participants’ perceived muscularity and the muscularity they ideally wished to possess (Leit et al., 2002). Experimental exposure to muscular and hypermuscular male images has also been linked with body-image responses, particularly among men with pre-existing muscularity or body-dissatisfaction concerns (Arbour and Martin Ginis, 2006). Contemporary fitness-role-model content may therefore present several overlapping standards—including thinness, muscularity, leanness, athletic competence, and disciplined lifestyle practices—against which students may evaluate their own bodies.

The present study focuses specifically on social physique anxiety, rather than general body dissatisfaction. Social physique anxiety refers to anxiety experienced when individuals anticipate that their physiques may be observed or negatively evaluated by others (Hart et al., 1989). Body dissatisfaction concerns individuals’ evaluations of their own bodies more broadly, whereas social physique anxiety emphasizes anticipated interpersonal evaluation and self-presentational concern. Fitness-role-model content may be particularly relevant to this form of anxiety because social media makes bodies, training outcomes, progress photographs, and physical performance publicly visible within an environment where appearance can be continuously viewed and evaluated. Repeated exposure to highly valued fitness bodies may therefore be associated with greater concern regarding whether one’s own physique meets visible social or personally valued standards. Accordingly:

*H1*: Exposure to fitness role models on social media is positively associated with social physique anxiety.

Social comparison theory provides the primary theoretical explanation for why fitness-role-model exposure may be associated with body comparison. Festinger (1954) proposed that individuals evaluate their attributes and abilities through comparison with others, particularly when objective standards are unavailable or ambiguous. Judgments concerning whether a body is sufficiently fit, lean, muscular, athletic, or attractive rarely have a single objective criterion. Visibly fit or athletic individuals can therefore provide salient reference targets. When such targets are perceived as possessing more desirable physiques, greater fitness progress, or other valued attributes, the comparison is upward.

A substantial body of body-image research supports the relevance of this process. In an experimental study, Tiggemann and Slater (2004) found that viewing appearance-focused music videos featuring thin women increased both social comparison and body dissatisfaction; comparison processing statistically mediated the association between experimental condition and body dissatisfaction. Myers and Crowther’s (2009) meta-analysis of 156 studies and 189 effect sizes similarly demonstrated a consistent association between social comparison and greater body dissatisfaction. Evidence from naturalistic settings also supports this relationship: Myers et al. (2012) found that naturally occurring upward appearance comparisons were associated with greater body-image disturbance among undergraduate women. The same process has subsequently been demonstrated in fitness-oriented social media. Tiggemann and Zaccardo (2015) found that state appearance comparison mediated the negative effect of fitspiration exposure on body-image outcomes, while a systematic review of experimental social-networking-site studies identified appearance comparison as an important process linking exposure to idealized imagery with adverse body-image outcomes (Fioravanti et al., 2022).

These findings provide a theoretical and empirical basis for extending appearance-comparison research to broader exposure to fitness role models on social media. Rather than focusing only on a single experimentally presented image, the present study considers repeated encounters with fitness-related targets across routine social media use. Upward body comparison is defined here as the tendency to compare one’s body shape, proportions, muscularity, body composition, fitness progress, or physical attractiveness with fitness-related targets perceived as physically superior. Accordingly:

*H2*: Exposure to fitness role models on social media is positively associated with upward body comparison.

Social comparison, however, is conceptually distinct from body self-discrepancy. Upward body comparison is externally referenced: it concerns how an individual evaluates their body relative to another person who is perceived as superior on relevant physical attributes. Body self-discrepancy is internally referenced: it concerns the perceived distance between one’s actual body and a personally valued ideal body state. Self-discrepancy theory proposes that individuals maintain representations of their actual self and personally meaningful self-guides and that discrepancies between these representations are associated with particular forms of emotional vulnerability (Higgins, 1987). In the body-image domain, the Body-Image Ideals Questionnaire operationalized this perspective by assessing both perceived discrepancy from personal physical ideals and the importance attached to those ideal attributes (Cash and Szymanski, 1995). A discrepancy is therefore likely to be more psychologically meaningful when the relevant attribute is strongly valued.

Although social comparison and body self-discrepancy are theoretically distinguishable, they may be related. An upward comparison target can provide a concrete representation of a body state that an individual regards as desirable, thereby making differences between the current and preferred body more salient. Prior research demonstrates that comparison and self-discrepancy can operate jointly in responses to idealized media, although it does not establish the specific ordering tested in the present study. Bessenoff (2006), for example, found that social comparison mediated adverse responses to thin-ideal media exposure, whereas body-image self-discrepancy moderated this mediation. Thus, self-discrepancy was treated as an individual-difference moderator rather than as a mediator following social comparison. Experimental research with muscular male imagery also found that exposure could increase perceived discrepancy between men’s actual and ideal muscularity (Leit et al., 2002). Building on these findings, the present study tests the theoretically extended proposition that stronger upward body comparison is positively associated with greater perceived body self-discrepancy. Accordingly:

*H3*: Upward body comparison is positively associated with body self-discrepancy.

Body self-discrepancy may also be relevant to social physique anxiety. When individuals perceive a substantial difference between their current body and a personally valued ideal, situations involving observation or evaluation of the body may become more psychologically salient. The original Body-Image Ideals Questionnaire showed that body-image discrepancy and ideal importance were associated with broader aspects of psychosocial adjustment, including social anxiety (Cash and Szymanski, 1995). More directly, Woodman and Steer (2011) demonstrated that body self-discrepancies involving ideal, ought, and feared body states were related to women’s social physique anxiety. These findings support the relevance of discrepancies between actual and valued body states to anxiety concerning the social evaluation of the physique. Accordingly:

*H4*: Body self-discrepancy is positively associated with social physique anxiety.

The preceding theoretical arguments also suggest two specific indirect associations. First, fitness-role-model exposure may be associated with social physique anxiety through upward body comparison. Idealized media research indicates that exposure can be associated with greater comparison processing and that such comparison is relevant to negative body evaluation (Fioravanti et al., 2022; Tiggemann and Slater, 2004; Tiggemann and Zaccardo, 2015). In a fitness-role-model context, repeated comparison with more desirable bodies may heighten awareness of external standards against which one’s own physique could be judged. Accordingly:

*H5*: Exposure to fitness role models on social media is indirectly associated with social physique anxiety through upward body comparison.

Second, fitness-role-model exposure may be associated with social physique anxiety through body self-discrepancy. Idealized fitness bodies may make personally valued body standards more salient, and experimental evidence indicates that exposure to muscular imagery can be associated with greater perceived actual–ideal discrepancy (Leit et al., 2002). Because greater body self-discrepancy is also relevant to social physique anxiety (Woodman and Steer, 2011), body self-discrepancy may account for an additional indirect association between exposure and social physique anxiety. Accordingly:

*H6*: Exposure to fitness role models on social media is indirectly associated with social physique anxiety through body self-discrepancy.

Finally, the two constructs can be incorporated into a theoretically ordered indirect-effect model. Within this model, exposure to fitness role models is associated with upward body comparison, upward body comparison is associated with body self-discrepancy, and body self-discrepancy is associated with social physique anxiety. Social comparison theory explains the relevance of external fitness targets as evaluative reference points, whereas self-discrepancy theory explains the psychological significance of perceived gaps between actual and personally valued ideal body states. The proposed ordering therefore integrates externally referenced comparison with internally referenced self-evaluation without treating the two constructs as conceptually interchangeable. Accordingly:

*H7*: Exposure to fitness role models on social media is indirectly associated with social physique anxiety through the theoretically ordered combination of upward body comparison and body self-discrepancy.

H7 concerns the theoretical ordering of statistical paths rather than an empirically established temporal process. Because the present study uses cross-sectional data, the model cannot determine whether upward body comparison temporally precedes body self-discrepancy, exclude reverse or reciprocal associations, or establish causal mediation.

The present study examines these hypotheses among college students and makes three related contributions. First, it extends established research on idealized media and body evaluation to the specific context of social-media fitness role models and to social physique anxiety as a socially evaluative outcome. Second, it brings social comparison theory and self-discrepancy theory into a single model while maintaining the conceptual distinction between externally referenced upward comparison and internally referenced body self-discrepancy. Third, exposure to fitness role models is conceptualized as a higher-order construct comprising passive browsing and active participation, thereby distinguishing incidental or platform-facilitated exposure from more intentional engagement with fitness-related content. The study does not assume that these two dimensions have identical independent associations with the outcome; rather, they are treated as complementary modes through which fitness-role-model exposure occurs.

Importantly, the article reports both a preliminary measurement phase and a formal hypothesis-testing phase. Before the formal survey, the questionnaire underwent expert evaluation and pilot testing. The pilot study retained 162 valid responses and was used to examine preliminary item performance, internal consistency, and dimensional structure of the four core measures. The subsequent formal study used 528 valid responses to evaluate the measurement model and test the hypothesized structural and indirect associations (Table 1).

**Table 1:** Hypotheses.

Serial number	Hypotheses
H1	*Exposure to fitness role models on social media is positively associated with social physique anxiety.*
H2	*Exposure to fitness role models on social media is positively associated with upward body comparison.*
H3	*Upward body comparison is positively associated with body self-discrepancy.*
H4	*Body self-discrepancy is positively associated with social physique anxiety.*
H5	*Exposure to fitness role models on social media is indirectly associated with social physique anxiety through upward body comparison.*
H6	*Exposure to fitness role models on social media is indirectly associated with social physique anxiety through body self-discrepancy.*
H7	*Exposure to fitness role models on social media is indirectly associated with social physique anxiety through the theoretically ordered combination of upward body comparison and body self-discrepancy.*

H = hypothesis. The hypotheses were tested using structural equation modeling and bootstrap mediation analysis.

## 2 Materials and methods

### 2.1 Participants and procedure

This study employed a cross-sectional online questionnaire design to examine the associations among exposure to fitness role models on social media, upward body comparison, body self-discrepancy, and social physique anxiety among college students, as well as the hypothesized indirect-effect model.

Participants were eligible for inclusion if they met the following criteria: (a) they were aged 18 years or older; (b) they were currently enrolled as college students; (c) they were studying at a university in Yunnan Province; and (d) they had used social media during the preceding month.

The questionnaire was administered online. Before completing the questionnaire, participants were informed of the purpose of the study, the voluntary nature of participation, the anonymity and confidentiality of their responses, and their right to withdraw at any time. No personally identifiable information, including names, student identification numbers, telephone numbers, or identity card numbers, was collected. The questionnaire comprised screening questions, demographic information, social media use and exercise-related questions, and four core measures assessing exposure to fitness role models on social media, upward body comparison, body self-discrepancy, and social physique anxiety.

The formal survey produced 560 questionnaires. Before statistical analysis, the responses were screened according to the predefined eligibility and data-quality criteria. Of the 560 questionnaires, 32 were excluded. Specifically, [*n* = 5] were excluded because the respondents did not meet the eligibility criteria, [*n* = 3] because valid informed consent was not provided, [*n* = 10] because the attention-check item was answered incorrectly, [*n* = 7] because the questionnaire was completed in an unreasonably short period of time, [*n* = 3] because responses to one or more core measures were incomplete, [*n* = 2] because implausible height or weight values were reported, and [*n* = 2] because the responses showed highly patterned or internally inconsistent answering. After screening, 528 valid questionnaires were retained, corresponding to a valid response rate of 94.29%.

Of the 528 valid participants, 428 were aged 22 years or younger (81.1%), and 100 were older than 22 years (18.9%). Regarding gender, 257 participants identified as male (48.7%), 184 as female (34.8%), 59 as non-binary or another gender identity (11.2%), and 28 preferred not to disclose their gender (5.3%). Undergraduate students accounted for 67.0% of the sample, followed by master’s students (23.1%), junior college students (7.8%), and doctoral students (2.1%).

Regarding body mass index, 344 participants were categorized in the 18–24 group (65.2%), 73 in the ≤18 group (13.8%), and 111 in the >24 group (21.0%). Most participants reported using social media for at least 2 h per day, and the most frequently used platforms included Douyin, Xiaohongshu, WeChat Official Accounts/Channels, Weibo, Bilibili, and Keep. Most participants also reported engaging in physical exercise at least twice per week. The sample therefore included students with varied demographic, social media use, and exercise-related backgrounds; however, it should not be interpreted as nationally representative.

### 2.2 Instrument development, translation, and contextual adaptation

The four core measures followed different development and adaptation procedures. Exposure to fitness role models on social media was assessed using a context-specific measure developed for the present study with reference to established English-language frameworks of passive and active social media use and fitness-related social media behavior. Upward body comparison was assessed using a translated and contextually adapted version of an established English-language appearance-comparison measure. Body self-discrepancy was operationalized according to the measurement logic of the Body-Image Ideals Questionnaire rather than through the direct translation of its original items. Social physique anxiety was assessed using a seven-item measure based on existing Chinese-language applications and psychometric evidence.

#### 2.2.1 Translation and cultural adaptation

The translation procedure was applied to the measure of exposure to fitness role models on social media and the measure of upward body comparison. For the exposure measure, the English-language source materials and relevant item formulations used in developing the preliminary measure were translated into Chinese. For the upward body comparison measure, the original English-language items were translated before being adapted to the context of fitness role models on social media.

The relevant English-language materials were independently translated into Chinese by bilingual researchers familiar with psychological measurement and sport- and fitness-related terminology. The independently produced Chinese versions were compared, and differences in terminology, sentence structure, and construct meaning were discussed to produce a reconciled Chinese version. Particular attention was given to expressions concerning passive browsing, active engagement, physique, muscularity, body-fat status, physical attractiveness, and fitness progress because these expressions could carry different meanings across linguistic and cultural contexts.

A separate bilingual researcher who had not participated in the forward translation and had not reviewed the original English wording then back-translated the reconciled Chinese items into English. The research team compared the back-translated version with the English-language source materials and revised the Chinese wording when conceptual or semantic inconsistencies were identified. The purpose of this procedure was to preserve the intended construct meaning rather than to achieve literal word-for-word equivalence.

The resulting Chinese items were subsequently submitted to an expert panel for assessment of content relevance, linguistic clarity, and cultural appropriateness. Expert comments were used to clarify wording and reduce contextual ambiguity without changing the intended construct meaning, item function, response format, or scoring method. The detailed expert-evaluation and pilot-testing procedures are presented in Section 2.3. The translation procedure was not applied to body self-discrepancy, which was operationalized according to an established measurement logic, or to social physique anxiety, for which items based on existing Chinese-language applications were used.

#### 2.2.2 Exposure to fitness role models on social media

Exposure to fitness role models on social media was assessed using a context-specific measure developed for the present study. The development of the measure was informed by the Passive and Active Facebook Use Measure, which distinguishes passive content consumption from active social media engagement (Gerson et al., 2017), and by Kim’s (2022) research on fitness-related social media postings among fitness app users. The English-language conceptual descriptions and relevant item formulations drawn from these sources were translated into Chinese and subsequently adapted to the context of college students’ exposure to fitness role models across contemporary social media platforms. The measure was therefore not treated as a direct translation of a single existing scale but as a translated and contextually developed instrument based on established measurement frameworks.

For the purposes of the present study, fitness role models included fitness bloggers, exercise content creators, fitness influencers, athletic peers, college students who shared fitness-related content, and ordinary users who publicly displayed their physique, training routines, exercise performance, exercise data, diet-management practices, body transformations, outdoor exercise, traditional sports, or other fitness-related lifestyles.

The preliminary measure contained 12 items representing two proposed dimensions: passive browsing and active participation. Passive browsing was assessed using six items that captured the extent to which participants unintentionally encountered fitness-related content through recommendation pages, trending pages, content feeds, or routine social media browsing. Active participation was assessed using six items that captured the extent to which participants actively searched for, followed, watched, saved, or interacted with fitness-role-model content or adjusted their training, diet, sleep, or body-management practices in response to such content.

Items were rated on a five-point frequency scale ranging from 1 (“*never*”) to 5 (“*always*”). Mean scores were calculated separately for passive browsing and active participation, with higher scores indicating more frequent exposure to fitness role models on social media. Passive browsing and active participation were specified as two first-order dimensions of the broader exposure construct.

#### 2.2.3 Upward body comparison

Upward body comparison was assessed using a Chinese version translated and contextually adapted from the Upward Physical Appearance Comparison Scale developed by O’Brien et al. (2009). The original measure was designed to assess individuals’ tendency to compare their physical appearance with others perceived as more attractive or physically ideal. Following translation and back-translation, the comparison target and item context were adapted so that the items referred specifically to fitness role models encountered on social media.

The adapted items assessed the extent to which participants compared their body shape, physique, body proportions, muscularity, body lines, body-fat status, fitness progress, and perceived physical attractiveness with those of fitness role models. The contextual modifications were intended to specify the comparison domain and target without changing the underlying meaning of upward physical-appearance comparison.

The adapted measure contained eight items. A sample item was: “When I see fitness role models on social media, I unconsciously compare my body with theirs.” Participants responded using a five-point Likert scale ranging from 1 (“*strongly disagree*”) to 5 (“*strongly agree*”). The mean score of the eight items was calculated, with higher scores indicating a stronger tendency to engage in upward body comparison when encountering fitness role models on social media.

#### 2.2.4 Body self-discrepancy

Body self-discrepancy was operationalized according to the measurement logic of the Body-Image Ideals Questionnaire developed by Cash and Szymanski (1995). Rather than treating the measure as a direct item-by-item translation of the original questionnaire, the present study retained its central measurement logic by considering both the perceived discrepancy between an individual’s actual and ideal body attributes and the personal importance assigned to those ideal attributes. This approach was consistent with the proposed conceptualization, which concerned both the perceived distance between participants’ current and ideal bodies and the extent to which the relevant ideal attributes were personally valued.

Participants were first instructed to imagine their personally endorsed ideal body state. The instructions emphasized that the ideal body did not refer to a standard imposed by social media or mass-aesthetic norms but to a body state that participants personally valued and hoped to approach. The ideal body could be associated with health, strength, posture, appearance, lifestyle, exercise ability, ethnic-cultural experience, or personal growth.

Participants subsequently rated 10 body attributes in two stages. First, they rated the perceived discrepancy between their actual and ideal body for each attribute. Second, they rated the personal importance of the corresponding ideal body attribute. The 10 attributes were height; body weight or body-shape heaviness; body proportion or overall posture; skin condition or skin tone; facial features; muscularity or strength; body lines or firmness; upper-body shape; physical strength and endurance; and body coordination or exercise performance.

Actual–ideal body discrepancy was rated on a four-point scale from 1 to 4, with higher scores indicating greater perceived discrepancy. The importance of ideal body attributes was also rated on a four-point scale from 1 to 4, with higher scores indicating greater personal importance. The actual–ideal discrepancy score and the attribute-importance score were calculated separately as the mean scores of their corresponding 10 ratings. The two components were retained to represent the measurement structure of body self-discrepancy.

#### 2.2.5 Social physique anxiety

Social physique anxiety was assessed using a seven-item version of the Social Physique Anxiety Scale originally developed by Hart et al. (1989). Unlike the first two measures, the present study did not newly translate the original English-language scale. Instead, the items were based on existing Chinese-language applications and psychometric evidence and were used in a form adapted to the Chinese college-student context.

Participants were asked to indicate the extent to which each statement described their actual feelings about their body, physique, or body shape. A sample item was: “If I know that others are looking at or evaluating my body shape, I feel uneasy.” Responses were rated on a five-point scale ranging from 1 (“*strongly inconsistent*”) to 5 (“*strongly consistent*”). The mean score of the seven items was calculated, with higher scores indicating higher social physique anxiety.

### 2.3 Expert evaluation and pilot testing

#### 2.3.1 Expert evaluation

Following the initial translation and contextual adaptation procedures described in Section 2.2, the first version of the questionnaire was submitted for expert evaluation. The evaluated questionnaire included the translated and contextually adapted measures of exposure to fitness role models on social media and upward body comparison, the body self-discrepancy measure operationalized according to the Body-Image Ideals Questionnaire framework, and the seven-item social physique anxiety measure based on existing Chinese-language applications.

The expert panel consisted of three experts with backgrounds in communication studies, sport science, and psychology, respectively. Each item was assessed in terms of content relevance, linguistic clarity, and cultural appropriateness. The item-level content validity index (I-CVI) was calculated as the proportion of experts assigning a relevance rating of 3 or higher among those who provided a valid relevance rating for the corresponding item. Missing ratings were not imputed and were excluded from the denominator of the relevant item-level calculation. The scale-level average content validity index (S-CVI/Ave) was calculated as the mean of the I-CVI values across all items, whereas the scale-level universal-agreement index (S-CVI/UA) represented the proportion of items with an I-CVI of 1.00.

The questionnaire obtained an S-CVI/Ave of 0.93 and an S-CVI/UA of 0.85. Of the 47 evaluated items, 40 obtained an I-CVI of 1.00. These findings provided evidence regarding the overall content relevance, linguistic clarity, and contextual appropriateness of the preliminary questionnaire. Because the number of valid ratings varied across several items, the item-level indices were interpreted together with the experts’ qualitative comments and the subsequent pilot results rather than being used as the sole basis for item deletion.

The experts generally considered the questionnaire understandable and culturally appropriate but identified several overlapping or contextually ambiguous items. For the exposure measure, wording was refined to distinguish incidental exposure from exposure focused on specific physique attributes and to differentiate training-related engagement from broader diet, sleep, and body-management practices. These revisions clarified item boundaries without changing the intended dimensions, response formats, or scoring procedures.

For upward body comparison, the expert comments were used to clarify the comparison target and domain. The wording was refined to emphasize comparisons involving body shape, physique, body proportions, muscularity, body lines, perceived strength, fitness progress, and overall physical attractiveness. M1-7 was explicitly restricted to fitness role models encountered on social media. M1-4 and M1-8 each obtained an I-CVI of 0.50 but were retained because they represented theoretically relevant aspects of physical-attractiveness comparison and repeated comparison frequency. These items were identified for particular attention during pilot testing.

For body self-discrepancy, M2-5 and the corresponding importance item M2I-5 each obtained an I-CVI of 0.50. For social physique anxiety, B1 and B4 obtained I-CVI values of 0.50, whereas B6 obtained an I-CVI of 0.67. These items were retained because they belonged to established measurement structures. The lower ratings were treated as indications for closer examination in the pilot item analysis, reliability analysis, and factor analysis rather than as sufficient evidence for deletion.

Expert feedback was used for linguistic clarification and contextual differentiation. The expert review was interpreted as evidence of content relevance, clarity, and contextual appropriateness rather than as evidence of reliability.

#### 2.3.2 Pilot testing

Following expert evaluation and the corresponding linguistic revisions, a revised preliminary questionnaire was produced and administered to college students who met the eligibility criteria specified in Section 2.1.

A total of 174 pilot questionnaires were returned. Twelve responses were excluded because of incomplete data or failure to satisfy the data-quality requirements, leaving 162 valid responses for the pilot analyses. The pilot study examined the internal consistency, item performance, and preliminary dimensional structure of the four core measures.

Internal consistency was assessed using Cronbach’s alpha coefficients, corrected item–total correlations, and alpha-if-item-deleted statistics. Particular attention was paid to items that received comparatively lower I-CVI values during expert evaluation, including M1-4, M1-8, M2-5, M2I-5, B1, B4, and B6. Their item–total correlations and contributions to scale reliability were examined before the final retention decisions were made.

The exposure measure showed a Cronbach’s alpha coefficient of 0.964, with corrected item–total correlations ranging from 0.759 to 0.846. Upward body comparison showed a Cronbach’s alpha coefficient of 0.963, with corrected item–total correlations ranging from 0.821 to 0.916. Body self-discrepancy showed a Cronbach’s alpha coefficient of 0.945, with corrected item–total correlations ranging from 0.535 to 0.779. Social physique anxiety showed a Cronbach’s alpha coefficient of 0.968, with corrected item–total correlations ranging from 0.700 to 0.928.

Although deleting B1 would have produced a higher alpha coefficient for social physique anxiety, B1 was retained because its corrected item–total correlation remained adequate and the item represented a theoretically relevant aspect of body-related distress. A marginal increase in Cronbach’s alpha was not considered sufficient justification for narrowing the content coverage of the measure.

Exploratory factor analysis was conducted to examine the preliminary dimensional structure. Before factor extraction, the Kaiser–Meyer–Olkin measure and Bartlett’s test of sphericity were used to assess the suitability of the pilot data for factor analysis.

For exposure to fitness role models on social media, the KMO value was 0.932, and a two-factor solution comprising active participation and passive browsing explained 81.784% of the cumulative rotated variance. For upward body comparison, the KMO value was 0.938, and a one-factor solution explained 79.850% of the variance, with factor loadings ranging from 0.861 to 0.939.

For body self-discrepancy, the KMO value was 0.899, and a two-factor solution comprising importance ratings and actual–ideal discrepancy ratings explained 77.185% of the cumulative rotated variance. For social physique anxiety, the KMO value was 0.937, and a one-factor solution explained 84.391% of the variance, with factor loadings ranging from 0.763 to 0.951. Bartlett’s tests were statistically significant for all four measures at *p < 0.001*. These results were treated as preliminary measurement evidence rather than as final evidence of construct validity.

#### 2.3.3 Final measurement decisions

Based on the combined consideration of theoretical relevance, expert comments, corrected item–total correlations, internal consistency results, and exploratory factor loadings, all items in the four core measures were retained for the formal survey.

Exposure to fitness role models on social media was specified as a higher-order construct comprising two first-order dimensions: passive browsing and active participation. This decision was consistent with both the conceptual structure of the measure and the two-factor solution identified in the pilot exploratory factor analysis.

Body self-discrepancy was specified as a higher-order construct comprising actual–ideal body discrepancy and the importance of ideal body attributes. These components were retained separately because the pilot results indicated that they were empirically distinguishable but theoretically connected.

Upward body comparison and social physique anxiety were retained as single-factor measures. The questionnaire resulting from these decisions constituted the final version used in the formal survey. Its measurement properties were subsequently examined in the formal-study sample through confirmatory factor analysis, construct reliability, convergent validity, and discriminant validity. These analyses were interpreted as initial measurement evidence supporting the proposed structure in the present samples rather than as comprehensive validation of a new instrument.

### 2.4 Final measures

#### 2.4.1 Exposure to fitness role models on social media

The final measure contained 12 items and comprised two first-order dimensions. Passive browsing was assessed using six items measuring unintentional exposure to fitness-related content through recommendation pages, trending pages, content feeds, or routine social media browsing. Active participation was assessed using six items measuring active searching, following, watching, saving, or interacting with fitness-role-model content and adjustments to training, diet, sleep, or body-management practices in response to such content.

Items were rated on a five-point frequency scale ranging from 1 (“*never*”) to 5 (“*always*”). Mean scores were calculated separately for passive browsing and active participation. Higher scores indicated more frequent exposure to fitness role models on social media. The two dimensions were specified as first-order factors of the higher-order exposure construct. For the descriptive and observed-score analyses, the overall exposure composite was calculated as the mean of the passive-browsing and active-participation dimension scores. Because the two dimensions contained equal numbers of items, this value was equivalent to the mean of all 12 exposure items.

#### 2.4.2 Upward body comparison

The final measure contained eight items assessing comparisons of body shape, physique, body proportions, muscularity, body lines, body-fat status, fitness progress, and perceived physical attractiveness with fitness role models encountered on social media. A sample item was: “When I see fitness role models on social media, I unconsciously compare my body with theirs.”

Responses were rated on a five-point Likert scale ranging from 1 (“*strongly disagree*”) to 5 (“*strongly agree*”). The mean score of the eight items was calculated, with higher scores indicating a stronger tendency toward upward body comparison.

#### 2.4.3 Social physique anxiety

Social physique anxiety was assessed using seven items measuring anxiety associated with the possibility that one’s body or physique might be observed or evaluated by others. A sample item was: “If I know that others are looking at or evaluating my body shape, I feel uneasy.”

Responses were rated on a five-point scale ranging from 1 (“*strongly inconsistent*”) to 5 (“*strongly consistent*”). The mean score of the seven items was calculated, with higher scores indicating higher social physique anxiety. No reverse scoring was applied.

#### 2.4.4 Body self-discrepancy

Body self-discrepancy was assessed through 10 body attributes: height; body weight or body-shape heaviness; body proportion or overall posture; skin condition or skin tone; facial features; muscularity or strength; body lines or firmness; upper-body shape; physical strength and endurance; and body coordination or exercise performance.

Participants first rated the discrepancy between their actual and personally ideal body for each attribute and then rated the personal importance of the corresponding ideal attribute. Both sets of ratings used four-point scales ranging from 1 to 4. Higher discrepancy scores indicated greater actual–ideal discrepancy, whereas higher importance scores indicated greater personal importance.

The mean scores of the 10 discrepancy ratings and the 10 importance ratings were calculated separately. In the primary measurement and structural models, these two components were specified as first-order factors of the higher-order body self-discrepancy construct. For the descriptive analyses and the primary observed-score sensitivity analysis, the overall body self-discrepancy composite was calculated as the arithmetic mean of the actual–ideal discrepancy component score and the attribute-importance component score; the two component scores were not multiplied or otherwise weighted.

To evaluate whether the observed indirect-association pattern depended on this primary operationalization, an alternative dimension-level multiplicative score was calculated for an additional sensitivity analysis. Specifically, the mean actual–ideal discrepancy score across the 10 body attributes was multiplied by the mean importance score across the corresponding 10 attributes:

$$\mathrm{BS}D_{\text{product}}=\text{Mean discrepancy}\times \text{Mean importance}$$

This dimension-level product score was used only in the supplementary sensitivity analysis and did not replace the higher-order latent specification used in the primary structural equation model.

#### 2.4.5 Descriptive and contextual variables

The questionnaire also collected demographic, social media use, exercise-related, and body-related information. Demographic variables included age, gender, education level, and grade. Height and weight were used to calculate body mass index. Social media variables included daily use duration and frequently used platforms, whereas exercise-related variables included weekly exercise frequency and average exercise duration.

Overall body satisfaction was assessed using one item ranging from 1 (“*very dissatisfied*”) to 5 (“*very satisfied*”). Participants’ main place of upbringing, ethnic-cultural background, and participation in traditional ethnic sports, ethnic dance, festival sports, outdoor mountain activities, or related physical activities during the preceding year were also recorded. These variables were primarily used to describe the sample and were not specified as core constructs in the hypothesized mediation model.

### 2.5 Data analysis

All formal analyses were conducted using the cleaned dataset of 528 valid responses described in Section 2.1. The scoring procedures for the four core measures followed the rules specified in Section 2.4.

Internal consistency was examined using Cronbach’s alpha coefficients and corrected item–total correlations. The suitability of the item data for factor analysis was assessed using the Kaiser–Meyer–Olkin measure and Bartlett’s test of sphericity.

Confirmatory factor analysis was conducted to evaluate the formal measurement model. Exposure to fitness role models on social media was specified as a higher-order construct comprising passive browsing and active participation. Body self-discrepancy was specified as a higher-order construct comprising actual–ideal body discrepancy and the importance of ideal body attributes. Upward body comparison and social physique anxiety were specified as single-factor constructs.

Model fit was evaluated using the chi-square-to-degrees-of-freedom ratio (χ^2^/df), root mean square residual (RMR), goodness-of-fit index (GFI), adjusted goodness-of-fit index (AGFI), incremental fit index (IFI), Tucker–Lewis index (TLI), comparative fit index (CFI), and root mean square error of approximation (RMSEA). Construct reliability and convergent validity were examined using standardized factor loadings, composite reliability, and average variance extracted. Discriminant validity was assessed using both the Fornell–Larcker criterion and the heterotrait–monotrait ratio of correlations (HTMT). For the Fornell–Larcker assessment, the square root of the AVE for each construct was compared with its correlations with the other constructs. HTMT was additionally examined as a complementary assessment of discriminant validity, with 0.85 used as the more conservative reference criterion (Henseler et al., 2015).

Because all four core constructs were assessed using self-report measures at the same time point, common method bias was examined using both Harman’s single-factor test and confirmatory factor analysis. For Harman’s test, all measurement items were entered into an unrotated exploratory factor analysis, and the variance explained by the first unrotated factor was examined.

For the CFA assessment of common method bias, a one-factor model was specified in which all measurement items loaded onto a single latent factor. The fit of this one-factor model was compared with that of the hypothesized measurement model containing the four theoretically distinct constructs and their specified dimensional structures. The comparison was based on χ^2^/df, RMR, GFI, AGFI, IFI, TLI, CFI, and RMSEA. A substantially poorer fit of the one-factor model relative to the hypothesized measurement model would indicate that a single common factor did not adequately account for the covariance among all measurement items. This CFA comparison was used as a supplementary assessment and was interpreted together with Harman’s single-factor test rather than as definitive evidence that common method bias was absent.

Descriptive statistics were calculated for the principal variables, including means, standard deviations, minimum and maximum values, skewness, and kurtosis. Pearson correlation analysis was used to examine the bivariate associations among exposure to fitness role models on social media, upward body comparison, body self-discrepancy, and social physique anxiety.

The hypothesized indirect-effect model was tested using latent-variable structural equation modeling. Exposure to fitness role models on social media was specified as the independent variable, upward body comparison as the first theoretically ordered mediator, body self-discrepancy as the second theoretically ordered mediator, and social physique anxiety as the outcome. The model additionally included direct paths from age, gender, BMI, educational background, grade, weekly exercise frequency, body satisfaction, participation in traditional, outdoor, or related physical activities, ethnicity, and main place of upbringing to social physique anxiety. These covariate paths were estimated simultaneously with the structural paths among the four core constructs. These variables were included as outcome-stage predictors of social physique anxiety and were not intended to constitute comprehensive confounder adjustment for all associations involving the two mediators. Bootstrap analysis with 5,000 resamples was used to estimate the total association, direct association, specific indirect associations, and the theoretically ordered indirect association. An indirect association was considered statistically supported when its 95% bootstrap confidence interval did not include zero.

Two supplementary unadjusted observed-score sensitivity analyses were conducted using PROCESS Model 6 with 5,000 bootstrap resamples. The first used the primary observed composite scores to examine whether the general pattern of indirect associations was reproduced without latent-variable estimation. The second specifically examined sensitivity to the operationalization of body self-discrepancy by replacing the primary body self-discrepancy composite with the alternative dimension-level product score, calculated as the mean actual–ideal discrepancy score multiplied by the mean importance score. Exposure to fitness role models, upward body comparison, social physique anxiety, and the theoretically specified ordering were otherwise unchanged. Both analyses were treated as supplementary sensitivity analyses rather than as alternative primary models. All statistical tests were two-tailed, with the significance level set at *p* < 0.05.

## 3 Results

### 3.1 Measurement model assessment

The measurement properties of the four core constructs were evaluated using the formal-study sample. Internal consistency was examined first, followed by confirmatory factor analysis, construct reliability, convergent validity, and discriminant validity. The measurement model treated exposure to fitness role models on social media and body self-discrepancy as higher-order constructs, whereas upward body comparison and social physique anxiety were specified as single-factor constructs.

#### 3.1.1 Internal consistency

The four core measures demonstrated satisfactory internal consistency. The overall Cronbach’s alpha coefficient for exposure to fitness role models on social media was 0.947. Its two first-order dimensions, passive browsing and active participation, showed alpha coefficients of 0.953 and 0.944, respectively. The corrected item–total correlations ranged from 0.831 to 0.880 for passive browsing and from 0.791 to 0.868 for active participation.

The upward body comparison measure showed a Cronbach’s alpha coefficient of 0.906, with corrected item–total correlations ranging from 0.607 to 0.746. Body self-discrepancy showed an overall alpha coefficient of 0.927. The actual–ideal body discrepancy component and the importance of ideal body attributes component showed alpha coefficients of 0.939 and 0.928, respectively, with corrected item–total correlations ranging from 0.660 to 0.808 across the two components. Social physique anxiety showed an alpha coefficient of 0.893, with corrected item–total correlations ranging from 0.614 to 0.762.

All corrected item–total correlations exceeded 0.60, and deleting individual items did not produce a substantively improved internal-consistency coefficient. Therefore, all items were retained in the formal measurement model (Table 2).

**Table 2:** Internal consistency of the measures in the formal study.

Construct or dimension	Number of items	Cronbach’s α	Corrected item–total correlation range
Exposure to fitness role models on social media	12	0.947	—
Passive browsing	6	0.953	0.831–0.880
Active participation	6	0.944	0.791–0.868
Upward body comparison	8	0.906	0.607–0.746
Body self-discrepancy	20	0.927	—
Actual–ideal body discrepancy	10	0.939	0.680–0.808
Importance of ideal body attributes	10	0.928	0.660–0.771
Social physique anxiety	7	0.893	0.614–0.762

Cronbach’s α values and corrected item–total correlations are based on the formal-study sample. The higher-order constructs are also presented with their corresponding first-order components.

#### 3.1.2 Confirmatory factor analysis

Confirmatory factor analysis was conducted to evaluate the hypothesized measurement structure. Exposure to fitness role models on social media was represented by passive browsing and active participation as two first-order factors. Body self-discrepancy was represented by actual–ideal body discrepancy and the importance of ideal body attributes as two first-order components. Upward body comparison and social physique anxiety were each represented by a single first-order factor.

The measurement model yielded χ^2^ = 2435.139 and χ^2^/df = 2.378. The residual-based indices were RMR = 0.041 and RMSEA = 0.051. The incremental fit indices were IFI = 0.927, TLI = 0.923, and CFI = 0.927. GFI and AGFI were 0.835 and 0.818, respectively.

Taken together, the hypothesized measurement model showed an overall acceptable fit. The χ^2^/df value was below 3, RMR was below 0.05, and IFI, TLI, and CFI exceeded 0.90. RMSEA was close to 0.05 and remained within an acceptable range. However, GFI and AGFI were below the conventional 0.90 reference value; therefore, the model should not be described as demonstrating uniformly excellent fit across all indices. The measurement structure was retained for the subsequent reliability, validity, and structural-model analyses (Table 3).

**Table 3:** Fit indices of the hypothesized measurement model.

Fit index	Observed value	Reference value	Interpretation
χ^2^	2435.139	—	Sample-size-sensitive statistic
χ^2^/df	2.378	< 3.00	Acceptable
RMR	0.041	< 0.05	Acceptable
GFI	0.835	> 0.90	Below reference value
AGFI	0.818	> 0.90	Below reference value
IFI	0.927	> 0.90	Acceptable
TLI	0.923	> 0.90	Acceptable
CFI	0.927	> 0.90	Acceptable
RMSEA	0.051	Approximately 0.05	Acceptable

RMR, root mean square residual; GFI, goodness-of-fit index; AGFI, adjusted goodness-of-fit index; IFI, incremental fit index; TLI, Tucker–Lewis index; CFI, comparative fit index; RMSEA, root mean square error of approximation.

#### 3.1.3 Construct reliability and convergent validity

Construct reliability and convergent validity were evaluated using standardized factor loadings, composite reliability, and average variance extracted. At the higher-order level, passive browsing and active participation loaded on exposure to fitness role models on social media at 0.942 and 0.914, respectively. The higher-order exposure construct showed a composite reliability of 0.926 and an average variance extracted of 0.861. The passive-browsing dimension showed standardized item loadings ranging from 0.857 to 0.902, with CR = 0.954 and AVE = 0.777. The active-participation dimension showed item loadings ranging from 0.821 to 0.898, with CR = 0.944 and AVE = 0.738.

For upward body comparison, standardized loadings ranged from 0.636 to 0.787, with CR = 0.909 and AVE = 0.557. For social physique anxiety, standardized loadings ranged from 0.657 to 0.803, with CR = 0.894 and AVE = 0.547.

At the higher-order level, the two body self-discrepancy components had standardized loadings of 0.864 and 0.832. The higher-order body self-discrepancy construct showed CR = 0.837 and AVE = 0.719. The actual–ideal body discrepancy component showed standardized item loadings ranging from 0.699 to 0.841, with CR = 0.940 and AVE = 0.611. The importance of ideal body attributes component showed standardized item loadings ranging from 0.687 to 0.801, with CR = 0.929 and AVE = 0.568.

Across the measurement model, all standardized factor loadings exceeded 0.60, composite-reliability values ranged from 0.837 to 0.954, and AVE values ranged from 0.547 to 0.861. These results supported the construct reliability and convergent validity of the specified measurement model (Table 4).

**Table 4:** Standardized factor loadings, composite reliability, and average variance extracted.

Construct or dimension	Standardized loading range	CR	AVE
Exposure to fitness role models on social media	0.914–0.942	0.926	0.861
Passive browsing	0.857–0.902	0.954	0.777
Active participation	0.821–0.898	0.944	0.738
Upward body comparison	0.636–0.787	0.909	0.557
Body self-discrepancy	0.832–0.864	0.837	0.719
Actual–ideal body discrepancy	0.699–0.841	0.940	0.611
Importance of ideal body attributes	0.687–0.801	0.929	0.568
Social physique anxiety	0.657–0.803	0.894	0.547

CR, composite reliability; AVE, average variance extracted. For the two higher-order constructs, the standardized loading range refers to the loadings of the corresponding first-order components.

#### 3.1.4 Discriminant validity

Discriminant validity was evaluated using the Fornell–Larcker criterion. The square root of the AVE for exposure to fitness role models on social media was 0.928, compared with its latent correlations of 0.623 with upward body comparison, 0.522 with body self-discrepancy, and 0.499 with social physique anxiety. The square roots of the AVE for upward body comparison, body self-discrepancy, and social physique anxiety were 0.746, 0.848, and 0.740, respectively. Each diagonal value exceeded the corresponding interconstruct correlations.

Of particular relevance to the potential distinction between the two mediators, the latent correlation between upward body comparison and body self-discrepancy was 0.515. This value was lower than the square root of the AVE for both upward body comparison (0.746) and body self-discrepancy (0.848). The results therefore indicated that the two constructs were related but empirically distinguishable within the specified measurement model. The findings should not be interpreted as indicating that the constructs have no conceptual overlap; rather, they support treating them as separate latent constructs in the subsequent structural analysis.

Discriminant validity was further assessed using HTMT. The HTMT values among the four core constructs ranged from 0.465 to 0.615, and all were below the conservative 0.85 reference criterion. Of particular relevance to the distinction between the two theoretically ordered mediators, the HTMT value between upward body comparison and body self-discrepancy was 0.521. Together with the Fornell–Larcker results, these findings provided additional evidence that the four constructs were empirically distinguishable in the present sample (Table 5).

**Table 5:** Discriminant validity of the core constructs.

Construct	1	2	3	4
Panel A. Fornell–Larcker criterion				
1. Exposure to fitness role models on social media	0.928			
2. Upward body comparison	0.623	0.746		
3. Body self-discrepancy	0.522	0.515	0.848	
4. Social physique anxiety	0.499	0.465	0.515	0.740
Panel B. HTMT ratios				
1. Exposure to fitness role models on social media	—			
2. Upward body comparison	0.615	—		
3. Body self-discrepancy	0.558	0.521	—	
4. Social physique anxiety	0.509	0.465	0.484	—

Panel A reports the Fornell–Larcker assessment; diagonal values are the square roots of average variance extracted (AVE), and off-diagonal values are latent interconstruct correlations. Panel B reports heterotrait–monotrait ratios of correlations (HTMT). All HTMT values were below 0.85.

### 3.2 Common method bias assessment

Because the four core constructs were measured concurrently using self-report questionnaires, potential common method bias was assessed using two complementary procedures: Harman’s single-factor test and a confirmatory factor analysis comparing a single-factor model with the hypothesized measurement model. These analyses were used to evaluate whether the covariance among the measurement items was dominated by a single general factor. They were not interpreted as definitive evidence that common method bias was completely absent.

#### 3.2.1 Harman’s single-factor test

Harman’s single-factor test was conducted by entering all measurement items into an unrotated exploratory factor analysis. The analysis extracted five factors with eigenvalues greater than 1. The first unrotated factor had an eigenvalue of 15.688 and accounted for 33.379% of the total variance. The five extracted factors jointly accounted for 66.328% of the total variance.

Because the first unrotated factor did not account for a dominant proportion of the total item variance, the results did not indicate an obvious single-factor structure. However, Harman’s test was treated only as a preliminary diagnostic because it does not directly model method-related variance and cannot, by itself, rule out common method bias (Table 6).

**Table 6:** Harman’s single-factor test.

Factor	Eigenvalue	Variance explained (%)	Cumulative variance (%)
1	15.688	33.379	33.379
2	5.656	12.035	45.414
3	4.479	9.530	54.944
4	2.821	6.002	60.946
5	2.530	5.382	66.328

Values are based on the initial unrotated factor solution. The first unrotated factor accounted for 33.379% of the total variance.

#### 3.2.2 CFA single-factor model comparison

A confirmatory single-factor model was additionally estimated in which all measurement items were constrained to load onto one latent factor. The single-factor model showed poor fit: χ^2^ = 10,975.619, χ^2^/df = 10.615, RMR = 0.132, GFI = 0.337, AGFI = 0.277, IFI = 0.485, TLI = 0.460, CFI = 0.483, and RMSEA = 0.135.

These indices were substantially poorer than those of the hypothesized measurement model, which showed χ^2^/df = 2.378, RMR = 0.041, GFI = 0.835, AGFI = 0.818, IFI = 0.927, TLI = 0.923, CFI = 0.927, and RMSEA = 0.051. The large deterioration in model fit indicated that a single latent factor did not adequately reproduce the covariance structure among all measurement items.

Taken together, Harman’s single-factor test and the CFA model comparison did not support the interpretation that the observed item covariance was dominated by one general factor. Nevertheless, because all core variables were measured through self-report questionnaires at the same time point, the possibility of common method bias could not be completely excluded (Table 7).

**Table 7:** Comparison of the hypothesized measurement model and the single-factor model.

Model	χ^2^	χ^2^/df	RMR	GFI	AGFI	IFI	TLI	CFI	RMSEA
Hypothesized measurement model	2,435.139	2.378	0.041	0.835	0.818	0.927	0.923	0.927	0.051
Single-factor model	10,975.619	10.615	0.132	0.337	0.277	0.485	0.460	0.483	0.135

RMR, root mean square residual; GFI, goodness-of-fit index; AGFI, adjusted goodness-of-fit index; IFI, incremental fit index; TLI, Tucker–Lewis index; CFI, comparative fit index; RMSEA, root mean square error of approximation. The single-factor model loaded all measurement items onto one latent factor.

### 3.3 Descriptive statistics and correlations

#### 3.3.1 Descriptive statistics

Descriptive statistics were calculated for the four core constructs and the first-order components of the two higher-order constructs. All variables were based on 528 valid responses. The observed scores covered most or all of their respective response ranges, indicating sufficient variability for subsequent correlational and structural analyses.

For exposure to fitness role models on social media, passive browsing had a mean of 3.688 (*SD* = 0.944), whereas active participation had a mean of 3.575 (*SD* = 0.853). The overall exposure score had a mean of 3.632 (*SD* = 0.877). Upward body comparison had a mean of 3.626 (*SD* = 0.765), and social physique anxiety had a mean of 3.332 (*SD* = 0.880).

For body self-discrepancy, the mean score for actual–ideal body discrepancy was 2.661 (*SD* = 0.659), and the mean score for the importance of ideal body attributes was 2.910 (*SD* = 0.653). The overall body self-discrepancy score had a mean of 2.785 (*SD* = 0.536).

The absolute skewness values ranged from 0.097 to 0.778, and the absolute kurtosis values ranged from 0.100 to 0.442 across the four core constructs. The corresponding first-order components also showed relatively limited skewness and kurtosis. These results indicated that none of the observed scale scores showed a pronounced univariate distributional departure (Table 8).

**Table 8:** Descriptive statistics for the study variables.

Variable	*N*	Minimum	Maximum	*M*	*SD*	Skewness	Kurtosis
Passive browsing	528	1.000	5.000	3.688	0.944	−0.778	−0.119
Active participation	528	1.500	5.000	3.575	0.853	−0.561	−0.381
Exposure to fitness role models on social media	528	1.420	5.000	3.632	0.877	−0.700	−0.300
Upward body comparison	528	1.500	5.000	3.626	0.765	0.097	−0.186
Actual–ideal body discrepancy	528	1.000	4.000	2.661	0.659	0.311	0.248
Importance of ideal body attributes	528	1.000	4.000	2.910	0.653	−0.247	−0.289
Body self-discrepancy	528	1.000	4.000	2.785	0.536	0.258	0.100
Social physique anxiety	528	1.000	5.000	3.332	0.880	−0.521	0.442

M = mean; SD = standard deviation. Exposure to fitness role models on social media, upward body comparison, and social physique anxiety were measured on five-point scales. Body self-discrepancy and its two components were measured on four-point scales. The means should therefore not be compared directly across constructs with different response ranges.

#### 3.3.2 Bivariate correlations

Pearson correlation analysis was conducted to examine the bivariate associations among exposure to fitness role models on social media, upward body comparison, body self-discrepancy, and social physique anxiety. All six correlations were positive and statistically significant at *p* < 0.01.

Exposure to fitness role models on social media was positively correlated with upward body comparison (*r* = 0.569, *p* < 0.01), body self-discrepancy (*r* = 0.387, *p* < 0.01), and social physique anxiety (*r* = 0.470, *p* < 0.01). Thus, more frequent exposure was concurrently associated with stronger upward body comparison, greater perceived body self-discrepancy, and higher social physique anxiety.

Upward body comparison was positively correlated with body self-discrepancy (*r* = 0.351, *p* < 0.01) and social physique anxiety (*r* = 0.421, *p* < 0.01). Body self-discrepancy was also positively correlated with social physique anxiety (*r* = 0.326, *p* < 0.01).

The correlation between upward body comparison and body self-discrepancy was statistically significant but not sufficiently large to suggest that the two observed composite scores were interchangeable. These bivariate results were consistent with the proposed associations among the constructs and provided a basis for the subsequent structural-model analysis. However, because the correlations were estimated from cross-sectional data, they did not establish directionality or temporal ordering (Table 9).

**Table 9:** Bivariate correlations among the core constructs.

Variable	1	2	3	4
1. Exposure to fitness role models on social media	1			
2. Upward body comparison	0.569**	1		
3. Body self-discrepancy	0.387**	0.351**	1	
4. Social physique anxiety	0.470**	0.421**	0.326**	1

*N* = 528. Values are Pearson correlation coefficients based on observed composite scores. **p < 0.01, two-tailed.

### 3.4 Structural model with covariate paths to social physique anxiety

A structural equation model was estimated to examine the associations among exposure to fitness role models on social media, upward body comparison, body self-discrepancy, and social physique anxiety. The model additionally included direct paths from the specified covariates to social physique anxiety.

#### 3.4.1 Structural model fit

The structural model with covariate paths to social physique anxiety yielded χ^2^ = 3,946.347 and χ^2^/df = 2.581. The incremental fit indices were IFI = 0.918, TLI = 0.917, and CFI = 0.918, while RMSEA was 0.055. These values supported an overall acceptable level of model fit. However, RMR = 0.068 exceeded the more stringent reference value of 0.05, and GFI = 0.878 and AGFI = 0.860 were below the conventional 0.90 reference value. The model was therefore interpreted as showing acceptable rather than uniformly excellent fit (Table 10).

**Table 10:** Fit indices of the structural model with covariate paths to social physique anxiety.

Fit index	Observed value	Reference value	Interpretation
χ^2^	3,946.347	—	Sample-size-sensitive statistic
χ^2^/df	2.581	< 3.00	Acceptable
RMR	0.068	< 0.05	Above reference value
GFI	0.878	> 0.90	Below reference value
AGFI	0.860	> 0.90	Below reference value
IFI	0.918	> 0.90	Acceptable
TLI	0.917	> 0.90	Acceptable
CFI	0.918	> 0.90	Acceptable
RMSEA	0.055	< 0.08	Acceptable

RMR, root mean square residual; GFI, goodness-of-fit index; AGFI, adjusted goodness-of-fit index; IFI, incremental fit index; TLI, Tucker–Lewis index; CFI, comparative fit index; RMSEA, root mean square error of approximation.

#### 3.4.2 Structural associations among the core constructs

Within the specified structural model, exposure to fitness role models on social media showed a statistically significant positive association with upward body comparison (β = 0.622, *B* = 0.494, *SE* = 0.037, C.R. = 13.208, *p* < 0.001) and body self-discrepancy (β = 0.332, *B* = 0.151, *SE* = 0.036, C.R. = 4.246, *p* < 0.001).

Upward body comparison showed a statistically significant positive association with body self-discrepancy (β = 0.314, *B* = 0.180, *SE* = 0.046, C.R. = 3.895, *p* < 0.001). These results were consistent with the associations specified in the theoretically ordered model, although the cross-sectional data did not establish that the variables occurred in the proposed temporal sequence.

Social physique anxiety was positively associated with body self-discrepancy (β = 0.285, *B* = 0.485, *SE* = 0.152, C.R. = 3.186, *p* = 0.001), exposure to fitness role models on social media (β = 0.263, *B* = 0.204, *SE* = 0.048, C.R. = 4.254, *p* < 0.001), and upward body comparison (β = 0.131, *B* = 0.128, *SE* = 0.061, C.R. = 2.113, *p* = 0.035). Thus, all six structural associations among the core constructs reached statistical significance within the specified model (Table 11, Figure 1).

**Table 11:** Structural associations among the core constructs.

Structural path	β	*B*	*SE*	C.R.	*p*
Exposure to fitness role models → Upward body comparison	0.622	0.494	0.037	13.208	< 0.001
Exposure to fitness role models → Body self-discrepancy	0.332	0.151	0.036	4.246	< 0.001
Upward body comparison → Body self-discrepancy	0.314	0.180	0.046	3.895	< 0.001
Body self-discrepancy → Social physique anxiety	0.285	0.485	0.152	3.186	0.001
Exposure to fitness role models → Social physique anxiety	0.263	0.204	0.048	4.254	< 0.001
Upward body comparison → Social physique anxiety	0.131	0.128	0.061	2.113	0.035

β = standardized structural coefficient; B = unstandardized coefficient; SE, standard error; C.R. = critical ratio. The directional arrows represent the specification of the statistical model and should not be interpreted as evidence of causal or temporal direction.

**Figure 1:**
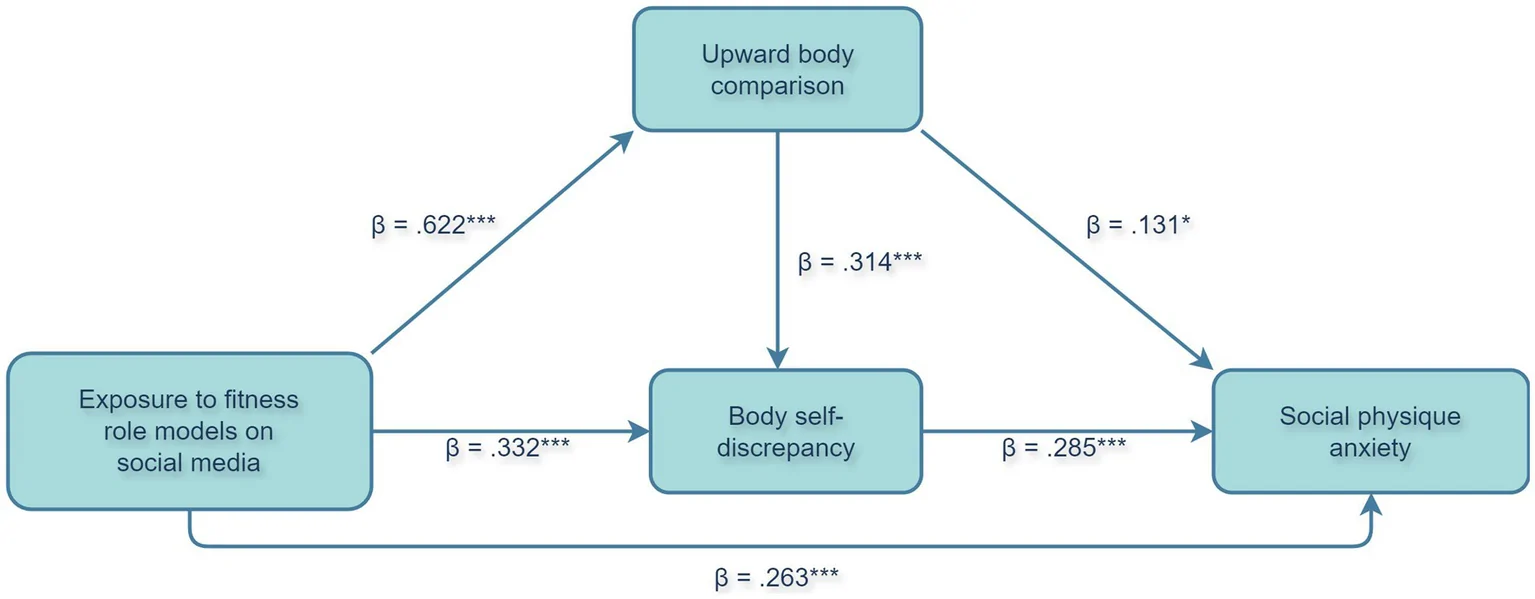
Structural model with covariate paths to social physique anxiety and standardized coefficients. Standardized structural coefficients are reported. Measurement indicators, error terms, and covariates are omitted for clarity. The directional arrows represent theoretically specified statistical paths and should not be interpreted as evidence of causal direction or temporal ordering. *p < 0.05, ***p ≤ 0.001.

#### 3.4.3 Associations involving the covariates

The model estimated direct paths from age, gender, BMI, educational background, grade, weekly exercise frequency, body satisfaction, participation in traditional, outdoor, or related physical activities, ethnicity, and growing location to social physique anxiety.

Weekly exercise frequency showed a statistically significant negative association with social physique anxiety (β = −0.121, *B* = −0.060, *SE* = 0.019, C.R. = − 3.100, *p* = 0.002). Participation in traditional, outdoor, or related physical activities showed a statistically significant positive association with social physique anxiety (β = 0.077, *B* = 0.051, *SE* = 0.026, C.R. = 1.982, *p* = 0.047). These coefficients indicate the associations observed within the specified multivariable model and should not be interpreted as causal effects.

Age (β = −0.051, *p* = 0.187), gender (β = 0.070, *p* = 0.072), BMI (β = 0.007, *p* = 0.856), educational background (β = 0.058, *p* = 0.134), grade (β = 0.068, *p* = 0.081), body satisfaction (β = −0.066, *p* = 0.088), ethnicity (β = 0.050, *p* = 0.198), and growing location (β = −0.045, *p* = 0.247) were not statistically associated with social physique anxiety at the predefined *p* < 0.05 level (Table 12).

**Table 12:** Associations between the covariates and social physique anxiety.

Covariate	β	*B*	*SE*	C.R.	*p*	Statistical result
Age	−0.051	−0.090	0.068	−1.318	0.187	Not significant
Gender	0.070	0.056	0.031	1.801	0.072	Not significant
BMI	0.007	0.008	0.045	0.181	0.856	Not significant
Educational background	0.058	0.067	0.045	1.497	0.134	Not significant
Grade	0.068	0.026	0.015	1.745	0.081	Not significant
Weekly exercise frequency	−0.121	−0.060	0.019	−3.100	0.002	Significant
Body satisfaction	−0.066	−0.048	0.028	−1.705	0.088	Not significant
Participation in traditional, outdoor, or related physical activities	0.077	0.051	0.026	1.982	0.047	Significant
Ethnicity	0.050	0.038	0.029	1.286	0.198	Not significant
Growing location	−0.045	−0.023	0.020	−1.158	0.247	Not significant

β = standardized structural coefficient; B = unstandardized coefficient; SE = standard error; C.R., critical ratio. Statistical significance was evaluated at *p* < 0.05.

### 3.5 Bootstrap analysis of indirect associations

Bootstrap analysis with 5,000 resamples was conducted to estimate the standardized total association, direct association, and three specific indirect associations between exposure to fitness role models on social media and social physique anxiety. An indirect association was considered statistically supported when its 95% bootstrap confidence interval did not include zero.

The standardized total association between exposure to fitness role models on social media and social physique anxiety was statistically different from zero (effect = 0.494, *SE* = 0.037, 95% CI [0.419, 0.547]). After upward body comparison and body self-discrepancy were included in the structural model, the standardized direct association also remained statistically different from zero (effect = 0.263, *SE* = 0.077, 95% CI [0.114, 0.349]). Thus, the association between exposure and social physique anxiety was not fully accounted for by the specified indirect paths.

The first specific indirect association, from exposure to fitness role models on social media to social physique anxiety through upward body comparison, was statistically different from zero (effect = 0.081, *SE* = 0.043, 95% CI [0.006, 0.145]). This result provided statistical support for H5 within the specified cross-sectional indirect-effect model.

The second specific indirect association, from exposure to fitness role models on social media to social physique anxiety through body self-discrepancy, was also statistically different from zero (effect = 0.095, *SE* = 0.058, 95% CI [0.027, 0.194]). This result provided statistical support for H6 within the specified model.

The theoretically ordered indirect association involving both mediators—exposure to fitness role models on social media → upward body comparison → body self-discrepancy → social physique anxiety—was statistically different from zero (effect = 0.056, *SE* = 0.030, 95% CI [0.014, 0.111]). The confidence interval did not include zero, providing statistical support for H7 within the hypothesized cross-sectional model.

Taken together, the bootstrap results identified statistically supported indirect associations through upward body comparison alone, body self-discrepancy alone, and the theoretically ordered combination of the two constructs. However, these findings do not establish that upward body comparison temporally preceded body self-discrepancy or that the variables unfolded in the sequence represented by the model. The directional paths reflect the theoretical specification of the statistical model rather than demonstrated causal or temporal ordering (Table 13).

**Table 13:** Bootstrap estimates of total, direct, and indirect associations.

Hypothesis or association	Statistical path	Standardized effect	*SE*	95% CI lower	95% CI upper	Result
Total association	Exposure to fitness role models on social media → Social physique anxiety	0.494	0.037	0.419	0.547	Significant
Direct association	Exposure to fitness role models on social media → Social physique anxiety	0.263	0.077	0.114	0.349	Significant
H5	Exposure → Upward body comparison → Social physique anxiety	0.081	0.043	0.006	0.145	Supported
H6	Exposure → Body self-discrepancy → Social physique anxiety	0.095	0.058	0.027	0.194	Supported
H7	Exposure → Upward body comparison → Body self-discrepancy → Social physique anxiety	0.056	0.030	0.014	0.111	Supported within the hypothesized cross-sectional model

SE, standard error; CI, confidence interval. An indirect association was considered statistically significant when its 95% bootstrap confidence interval did not include zero. The directional arrows reflect the theoretically specified statistical model and do not establish causal direction or temporal sequence.

### 3.6 Sensitivity analysis using the primary observed composite scores

A supplementary sensitivity analysis was conducted using PROCESS Model 6 with the observed composite scores of the four core constructs. Exposure to fitness role models on social media was entered as the independent variable, upward body comparison as the first mediator, body self-discrepancy as the second mediator, and social physique anxiety as the outcome. Bootstrap confidence intervals were estimated using 5,000 resamples at the 95% confidence level. Unlike the primary structural equation model, this analysis used observed composite scores and did not explicitly account for measurement error. This sensitivity analysis was conducted without covariates and was interpreted solely as a supplementary observed-score analysis rather than as an alternative primary model.

In the first regression equation, exposure to fitness role models on social media was positively associated with social physique anxiety (B = 0.472, *SE* = 0.039, *t* = 12.215, *p* < 0.001). The model explained 22.1% of the variance in social physique anxiety, *F* = 149.211, *p* < 0.001.

In the second equation, exposure was positively associated with upward body comparison (B = 0.496, *SE* = 0.031, *t* = 15.878, *p* < 0.001). This model explained 32.4% of the variance in upward body comparison, *F* = 252.108, *p* < 0.001.

In the third equation, both exposure to fitness role models on social media (B = 0.169, *SE* = 0.029, *t* = 5.726, *p* < 0.001) and upward body comparison (B = 0.136, *SE* = 0.034, *t* = 4.023, *p* < 0.001) were positively associated with body self-discrepancy. Together, the two variables explained 17.5% of the variance in body self-discrepancy, *F* = 55.612, *p* < 0.001.

In the final equation, exposure to fitness role models on social media (B = 0.303, *SE* = 0.047, *t* = 6.464, *p* < 0.001), upward body comparison (B = 0.231, *SE* = 0.053, *t* = 4.360, *p* < 0.001), and body self-discrepancy (B = 0.228, *SE* = 0.067, *t* = 3.392, *p* = 0.001) were each positively associated with social physique anxiety. The final equation explained 27.2% of the variance in social physique anxiety, *F* = 65.265, *p* < 0.001.

Bootstrap decomposition showed that the total association between exposure to fitness role models on social media and social physique anxiety was statistically significant (effect = 0.472, *SE* = 0.039, 95% CI [0.396, 0.547]). After the two observed mediator scores were included, the direct association remained statistically significant (effect = 0.303, *SE* = 0.047, 95% CI [0.211, 0.395]).

The total indirect association was 0.169 (*SE* = 0.035, 95% CI [0.098, 0.236]). The specific indirect association through upward body comparison was 0.115 (*SE* = 0.031, 95% CI [0.054, 0.176]). The indirect association through body self-discrepancy was 0.039 (*SE* = 0.016, 95% CI [0.010, 0.074]). The theoretically ordered indirect association through upward body comparison and body self-discrepancy was 0.015 (*SE* = 0.007, 95% CI [0.004, 0.032]). All bootstrap confidence intervals excluded zero (Tables 14, 15).

**Table 14:** Regression equations in the supplementary PROCESS Model 6 sensitivity analyses.

Outcome	Predictor	B	*SE*	*t*	*p*	*R* ^2^	*F*
Panel A. Primary observed composite scoring							
Social physique anxiety	Exposure to fitness role models on social media	0.472	0.039	12.215	< 0.001	0.221	149.211
Upward body comparison	Exposure to fitness role models on social media	0.496	0.031	15.878	< 0.001	0.324	252.108
Body self-discrepancy	Exposure to fitness role models on social media	0.169	0.029	5.726	< 0.001	0.175	55.612
	Upward body comparison	0.136	0.034	4.023	< 0.001		
Social physique anxiety	Exposure to fitness role models on social media	0.303	0.047	6.464	< 0.001	0.272	65.265
	Upward body comparison	0.231	0.053	4.360	< 0.001		
	Body self-discrepancy	0.228	0.067	3.392	0.001		
Panel B. Alternative dimension-level discrepancy × importance scoring							
Social physique anxiety	Exposure	0.472	0.039	12.215	< 0.001	0.221	149.211
Upward body comparison	Exposure	0.496	0.031	15.878	< 0.001	0.324	252.108
BSD product score	Exposure	0.868	0.174	4.993	< 0.001	0.158	49.113
	Upward body comparison	0.837	0.199	4.197	< 0.001		
Social physique anxiety	Exposure	0.300	0.046	6.479	< 0.001	0.281	68.177
	Upward body comparison	0.222	0.053	4.206	< 0.001		
	BSD product score	0.048	0.011	4.243	< 0.001		

Panel A uses the primary observed body self-discrepancy composite. Panel B uses the alternative dimension-level body self-discrepancy product score calculated as mean actual–ideal discrepancy × mean importance. Exposure = exposure to fitness role models on social media; BSD product score = alternative body self-discrepancy product score; B = unstandardized regression coefficient; SE = standard error. R^2^ and F refer to the complete regression equation for the corresponding outcome. Both analyses were unadjusted and used observed scores.

**Table 15:** Bootstrap decomposition of associations in the supplementary PROCESS sensitivity analyses.

Association	Effect	*SE*	95% CI lower	95% CI upper	Proportion of total association
Panel A. Primary observed composite scoring					
Total association	0.472	0.039	0.396	0.547	100.00%
Direct association	0.303	0.047	0.211	0.395	64.19%
Total indirect association	0.169	0.035	0.098	0.236	35.81%
Exposure → Upward body comparison → Social physique anxiety	0.115	0.031	0.054	0.176	24.36%
Exposure → Body self-discrepancy → Social physique anxiety	0.039	0.016	0.010	0.074	8.26%
Exposure → Upward body comparison → Body self-discrepancy → Social physique anxiety	0.015	0.007	0.004	0.032	3.18%
Panel B. Alternative dimension-level discrepancy × importance scoring					
Total association	0.472	0.039	0.396	0.547	100.00%
Direct association	0.300	0.046	0.209	0.391	63.56%
Total indirect association	0.172	0.035	0.105	0.242	36.44%
Exposure → Upward body comparison → SPA	0.110	0.031	0.051	0.172	23.31%
Exposure → BSD product → SPA	0.042	0.017	0.014	0.080	8.90%
Exposure → Upward body comparison → BSD product → SPA	0.020	0.009	0.006	0.040	4.24%

Panel A uses the primary observed body self-discrepancy composite. Panel B uses the alternative dimension-level product score calculated as mean actual–ideal discrepancy × mean importance. Exposure = exposure to fitness role models on social media; SPA = social physique anxiety; BSD product = alternative body self-discrepancy product score. Effect estimates are unstandardized. Bootstrap confidence intervals were estimated from 5,000 resamples. The proportions are descriptive and should not be interpreted as evidence of causal mediation. CI, confidence interval; SE, standard error.

The direct association represented 64.19% of the total association, whereas the combined indirect associations represented 35.81%. The indirect associations through upward body comparison, body self-discrepancy, and both mediators represented 24.36, 8.26, and 3.18% of the total association, respectively. These proportions are presented descriptively and should not be interpreted as evidence of causal mediation.

The unadjusted PROCESS analysis produced a broadly similar pattern of positive and statistically significant indirect associations. However, the numerical estimates should not be directly compared with those from the primary latent-variable model because the two analyses differed in both their treatment of measurement error and their inclusion of covariates.

### 3.7 Sensitivity analysis using an alternative dimension-level body self-discrepancy product score

An additional unadjusted PROCESS Model 6 sensitivity analysis examined whether the indirect-association pattern was sensitive to the operationalization of body self-discrepancy. In this analysis, body self-discrepancy was represented by the dimension-level product of the mean actual–ideal discrepancy score and the mean importance score. Exposure to fitness role models, upward body comparison, and social physique anxiety retained the same observed-score operationalizations as in Section 3.6.

Exposure to fitness role models was positively associated with upward body comparison (B = 0.496, SE = 0.031, t = 15.878, *p* < 0.001). In the equation predicting the alternative body self-discrepancy product score, both exposure (B = 0.868, SE = 0.174, t = 4.993, *p* < 0.001) and upward body comparison (B = 0.837, SE = 0.199, t = 4.197, *p* < 0.001) were positively associated with the product score. In the final equation predicting social physique anxiety, exposure (B = 0.300, SE = 0.046, t = 6.479, *p* < 0.001), upward body comparison (B = 0.222, SE = 0.053, t = 4.206, *p* < 0.001), and the alternative body self-discrepancy product score (B = 0.048, SE = 0.011, t = 4.243, *p* < 0.001) were positively associated with the outcome. The final equation explained 28.1% of the variance in social physique anxiety (R^2^ = 0.281, *F* = 68.177).

Bootstrap decomposition showed a total association of 0.472 (SE = 0.039, 95% CI [0.396, 0.547]) and a direct association of 0.300 (SE = 0.046, 95% CI [0.209, 0.391]). The total indirect association was 0.172 (SE = 0.035, 95% CI [0.105, 0.242]). The specific indirect association through upward body comparison was 0.110 (SE = 0.031, 95% CI [0.051, 0.172]), the indirect association through the alternative body self-discrepancy product score was 0.042 (SE = 0.017, 95% CI [0.014, 0.080]), and the theoretically ordered indirect association through upward body comparison and the alternative body self-discrepancy product score was 0.020 (SE = 0.009, 95% CI [0.006, 0.040]). All three specific indirect-association confidence intervals excluded zero.

## 4 Discussion

### 4.1 Principal findings and scope of inference

This study examined the associations among exposure to fitness role models on social media, upward body comparison, body self-discrepancy, and social physique anxiety among college students. In the latent-variable model that included the specified covariate paths to social physique anxiety, greater exposure to fitness role models was positively associated with stronger upward body comparison, greater body self-discrepancy, and higher social physique anxiety. Upward body comparison was also positively associated with body self-discrepancy and social physique anxiety, while body self-discrepancy was positively associated with social physique anxiety. Bootstrap analysis further identified statistically significant indirect associations through upward body comparison alone, body self-discrepancy alone, and the theoretically ordered combination of the two constructs. Because the direct association between exposure and social physique anxiety remained statistically significant after the two mediators were included, the association was not statistically accounted for by the specified indirect paths alone.

The supplementary analysis based on observed composite scores produced a broadly similar pattern of positive and statistically significant associations. However, the numerical estimates should not be directly compared with those from the primary latent-variable model because the two analyses differed in both their treatment of measurement error and their inclusion of covariates. The additional scoring sensitivity analysis yielded a broadly similar pattern when body self-discrepancy was alternatively represented by the dimension-level product of mean actual–ideal discrepancy and mean attribute importance. The bootstrap confidence intervals for the indirect association through this alternative body self-discrepancy score and for the theoretically ordered indirect association involving both upward body comparison and the product score excluded zero. Taken together, the supplementary analyses indicate that a broadly similar indirect-association pattern was observed under both the primary observed composite scoring and the alternative dimension-level product scoring of body self-discrepancy. Nevertheless, this additional analysis represents a scoring sensitivity check and does not establish that the alternative product score is a superior or fully validated measure of body self-discrepancy. More broadly, the findings suggest that exposure to fitness-role-model content may represent a psychologically relevant body-evaluative context for college students. Fitness-related social media can provide exercise information and health-oriented cues while simultaneously operating in a visual environment in which idealized bodies, athletic appearance, and physical transformation are salient (Tricás-Vidal et al., 2022; Wang et al., 2024; Xiao et al., 2025). The present results indicate that greater exposure to this content is associated not only with engagement in body comparison but also with anxiety concerning how one’s physique may be observed or evaluated by others.

These findings must nevertheless be interpreted within the limits of the cross-sectional design. The statistically significant indirect association involving upward body comparison and body self-discrepancy was consistent with the theoretically ordered model, but it did not establish that exposure temporally preceded upward comparison, that upward comparison subsequently preceded body self-discrepancy, or that these variables produced social physique anxiety. The directional arrows in the structural model represent theoretically specified statistical relations rather than demonstrated causal or temporal processes. Reverse or alternative associations remain plausible; for example, students with greater body self-discrepancy or higher social physique anxiety may attend more closely to fitness-role-model content. Accordingly, the findings should be understood as evidence of concurrent direct and indirect associations that are compatible with the proposed theoretical ordering, rather than as confirmation of a sequential psychological mechanism.

### 4.2 Fitness role model exposure, body evaluation, and social physique anxiety

In the structural model that included the specified covariate paths to social physique anxiety, exposure to fitness role models on social media was positively associated with social physique anxiety. One possible explanation is that fitness-related social media content frequently presents exercise through visible body transformation, athletic appearance, muscularity, leanness, and performative bodily display. A longitudinal content analysis of Instagram fitness posts found that female fitness-influencer content more frequently featured fit-ideal bodies and sexualized presentations than fitness-brand content, indicating that exercise-related communication may simultaneously convey appearance-based standards (Ahrens et al., 2022). Within such a visual environment, fitness role models may become salient reference points for evaluating one’s own physique. The present findings therefore suggest that more frequent exposure is associated with greater concern about how one’s body may be observed or evaluated by others. This interpretation remains tentative because the study did not directly analyze the specific content encountered by participants.

Upward body comparison represents one body-evaluative process associated with this exposure. Previous research has consistently linked fitspiration and idealized social media imagery with appearance comparison and adverse body-image outcomes. A systematic review reported that fitspiration exposure was associated with body dissatisfaction, physical appearance comparison, and negative mood (Jerónimo and Carraça, 2022). Similarly, a systematic review of experimental studies found that appearance comparison played an important explanatory role in the association between idealized social-networking images and negative body-image outcomes (Fioravanti et al., 2022). Pedalino and Camerini (2022) further found that upward comparison with influencers mediated the association between Instagram browsing and lower body appreciation among young women. Consistent with this literature, the present study showed that greater exposure to fitness role models was associated with stronger upward body comparison, and that the indirect association between exposure and social physique anxiety through upward body comparison was statistically significant. These results indicate that externally referenced body evaluation is relevant to the association under investigation, but they do not establish that exposure caused participants to engage in comparison.

Body self-discrepancy represents a related but conceptually distinct form of body evaluation. Upward body comparison concerns how individuals evaluate their bodies relative to another person perceived as physically superior, whereas body self-discrepancy concerns the perceived distance between one’s actual body and personally endorsed ideal body. Previous research has treated actual–ideal body discrepancy as an important indicator of body dissatisfaction and body-image disturbance. For example, Jakobek et al. (2024) examined discrepancies between actual and ideal body images among kinesiology students, while Liu et al. (2025) linked actual–ideal body-size discrepancy with body-size estimation processes among Chinese adolescent girls. In the present study, upward body comparison and body self-discrepancy were positively related, whereas both the Fornell–Larcker and HTMT results provided evidence that they were empirically distinguishable within the specified measurement model. The indirect association through body self-discrepancy was also statistically significant, suggesting that internal actual–ideal body evaluation provides explanatory information beyond externally referenced comparison alone.

The outcome examined in this study should also be distinguished from general body dissatisfaction. Social physique anxiety specifically concerns anxiety arising from the possibility that one’s physique may be observed or negatively evaluated by others. The use of the Social Physique Anxiety Scale in the present context is consistent with previous psychometric research among Chinese university students (Jin and Fung, 2021), as well as studies connecting negative physical self-evaluation and body dissatisfaction with social physique anxiety in student and physical-activity contexts (Jiang and Wang, 2025; Tsartsapakis et al., 2023). Taken together, the findings were consistent with a theoretically ordered model in which exposure to fitness role models was associated with externally referenced comparison, internally perceived actual–ideal discrepancy, and anxiety concerning social evaluation of the physique. However, the statistically significant indirect association involving both body-evaluation constructs does not demonstrate that they occurred in this temporal order.

### 4.3 Theoretical implications

Fitness-related social media research has frequently examined exercise motivation, physical activity intentions, and engagement with health-oriented content, whereas appearance-focused social media research has more commonly examined body dissatisfaction, appearance comparison, and related psychological outcomes. By focusing on fitness role models, the present study shows that the same social media context may be relevant to both health-oriented engagement and body-evaluative anxiety. Its contribution therefore lies in extending the study of fitness-related social media beyond behavioral engagement and situating it within a more specific account of how exposure is associated with concerns about the social evaluation of one’s physique. This contribution should be understood as an extension of existing research rather than evidence that fitness-role-model exposure has a uniform psychological effect.

Social comparison theory and self-discrepancy theory offer complementary explanations for the observed association pattern. Social comparison theory explains why physically idealized fitness role models may function as external reference targets, whereas self-discrepancy theory explains the relevance of perceived differences between one’s actual body and personally endorsed ideal body. Upward body comparison therefore represents an externally referenced evaluation, while body self-discrepancy represents an internally organized evaluation of actual and ideal body states. Their simultaneous inclusion in the model provides a more differentiated explanation than treating body evaluation as a single undivided process. The statistically significant indirect associations involving each construct, together with their demonstrated empirical distinctiveness, suggest that they provide complementary rather than interchangeable explanatory information.

The indirect association involving both constructs was consistent with the theoretical proposition that external comparison and internal self-evaluation may be connected. This interpretation aligns with broader evidence identifying appearance comparison as an important process in the association between idealized social media images and body-related outcomes (Fioravanti et al., 2022; Pedalino and Camerini, 2022). However, the theoretical ordering should not be interpreted as an observed temporal progression. The cross-sectional data cannot determine whether upward body comparison preceded body self-discrepancy, whether the two forms of body evaluation developed concurrently, or whether an alternative direction better represents their relationship. Accordingly, the theoretical contribution is the integration of two established body-evaluative constructs within one statistical model, rather than the demonstration of a definitive sequential psychological mechanism.

Exposure to fitness role models was represented as a higher-order construct comprising passive browsing and active participation. Rather than treating social media use as a single frequency-based variable, the study represented exposure as a higher-order construct comprising passive browsing and active participation. Passive browsing captured incidental or platform-facilitated encounters with fitness content through recommendation pages, trending pages, feeds, and routine social media use. Active participation captured intentional searching, following, viewing, saving, interaction, and the use of fitness-role-model content in training or body-management practices. These dimensions therefore represent distinct routes through which students encounter and engage with fitness role models, while remaining components of a broader exposure construct. The preliminary factor analysis and formal measurement model provided initial evidence consistent with this two-dimensional representation in the present samples.

This multidimensional conceptualization helps distinguish exposure to fitness-role-model content from undifferentiated measures of general social media use. Nevertheless, the structural model estimated the associations of the higher-order exposure construct rather than the separate associations of passive browsing and active participation. The findings therefore do not indicate that one form of exposure is more strongly associated with upward body comparison, body self-discrepancy, or social physique anxiety than the other. Future research should examine whether incidental, algorithmically facilitated exposure and intentional, behaviorally engaged exposure show different associations with body-evaluative outcomes.

Social physique anxiety should also be distinguished from broader body-image outcomes. Social physique anxiety concerns anticipated observation or negative evaluation of one’s physique by others, whereas body dissatisfaction refers more generally to negative evaluations of one’s own body. By linking fitness-role-model exposure with upward body comparison, body self-discrepancy, and social physique anxiety, the model provides a more focused account of how fitness-related social media experiences are associated with anxiety about bodily presentation in a social context.

### 4.4 Practical implications

The findings have implications for fitness communication and health promotion in university settings, although these implications should be interpreted in light of the correlational and cross-sectional design. Fitness-role-model content may be more appropriate when it presents exercise as a process involving physical function, health maintenance, skill development, and varied progress trajectories rather than implying that a narrow physique ideal is the expected outcome of exercise. This recommendation is consistent with evidence that fitness-related social media may support physical-activity engagement while fitspiration and idealized images are also associated with appearance comparison and body-image concerns (Jerónimo and Carraça, 2022; Tricás-Vidal et al., 2022).

For college physical education and health education, media-literacy programmes could incorporate greater attention to body comparison. Students may benefit from learning to identify selective self-presentation, idealized physique displays, transformation narratives, and appearance-centred fitness messaging. Such education could also encourage students to distinguish between exercise information that emphasizes health or physical function and content that implicitly presents one body type as a universal standard. However, the present findings do not establish that fitness-role-model content is inherently harmful or harmless. They indicate only that upward body comparison and body self-discrepancy are statistically relevant to the association between exposure and social physique anxiety. Whether similar content elicits different responses across students, and which individual or contextual characteristics shape comparison tendencies, remain open questions for future research.

For content creators and campus health communicators, the findings point to the value of reducing unnecessary comparison pressure in fitness messaging. Presenting diverse body types, training goals, ability levels, starting points, and rates of progress may help prevent exercise from being framed primarily as a means of attaining a single idealized appearance. Messages can also place greater emphasis on sustainable participation, enjoyment, competence, strength, and well-being. These recommendations are implications derived from the observed association pattern; the study did not directly test whether changing message design would reduce upward body comparison, body self-discrepancy, or social physique anxiety. Intervention studies are therefore needed before the effectiveness of these communication strategies can be established.

### 4.5 Limitations and future research

Several limitations should be considered when interpreting the findings. First, the cross-sectional design prevents conclusions about causal direction or temporal ordering. Although the hypothesized model specified exposure to fitness role models, upward body comparison, body self-discrepancy, and social physique anxiety in a theoretically ordered sequence, the data were collected at a single time point. It is therefore possible that students with greater body self-discrepancy or higher social physique anxiety attend more closely to fitness-role-model content, or that upward body comparison and body self-discrepancy develop concurrently rather than sequentially. The present study also did not formally compare the hypothesized model with reverse-direction or alternative-ordering models. Accordingly, the results indicate that the observed covariance pattern was compatible with the proposed model, not that this model represented the only plausible ordering of the constructs. Longitudinal, cross-lagged, experimental, and ecological momentary assessment designs are needed to examine temporal precedence and reciprocal associations.

Second, all core variables were assessed using self-report questionnaires administered within the same survey. Although Harman’s single-factor test and the poor fit of the CFA single-factor model indicated that one general factor did not adequately account for the covariance among the items, these procedures cannot completely exclude common method bias. Responses may also have been affected by recall error, social desirability, and differences in participants’ interpretation of their own social media behavior and body-related experiences. Future research could combine self-report measures with digital trace data, objective indicators of content exposure, behavioral measures of comparison, informant reports, or temporally separated measurement occasions. Experimental manipulation of fitness-role-model content would also help determine whether the observed association pattern is reproduced under more controlled conditions.

Third, the measurement evidence for exposure to fitness role models on social media should be regarded as preliminary. Expert evaluation, pilot exploratory factor analysis, formal confirmatory factor analysis, and internal-consistency estimates provided initial evidence consistent with a higher-order construct comprising passive browsing and active participation in the present samples. However, the measure has not yet been examined extensively in independent samples or through test–retest reliability, criterion validity, incremental validity, and measurement-invariance analyses. Moreover, the structural model estimated the association of the overall higher-order exposure construct rather than the separate associations of passive browsing and active participation. The present findings therefore cannot determine whether incidental, algorithmically facilitated browsing and intentional, behaviorally engaged participation have different relationships with body comparison or social physique anxiety. Future studies should validate the measure across populations and directly compare the two dimensions within structural models.

The operationalization of body self-discrepancy also warrants further evaluation. In the primary latent-variable model, actual–ideal body discrepancy and the importance attached to ideal body attributes were represented as two first-order components of a higher-order construct. The supplementary sensitivity analysis provided an initial comparison by using a dimension-level multiplicative score calculated as the product of the mean actual–ideal discrepancy score and the mean importance score, and the principal indirect-association pattern remained statistically supported under this alternative specification. However, this sensitivity score represents a dimension-level multiplicative operationalization and should not be interpreted as establishing the superiority of one scoring approach over another. Further research should compare alternative operationalizations and examine their criterion, predictive, and cross-group validity.

Fourth, the characteristics of the sample limit the generalizability of the findings. The results represent the college students included in the present study and should not be assumed to apply to all university students, adolescents, older adults, athletes, or social media users in other cultural and geographical contexts. Body ideals, platform-use patterns, fitness participation, and the social meaning attached to muscularity or thinness may vary across populations. Future research should use multi-site and more diverse samples and examine measurement and structural invariance across gender, region, educational level, exercise background, and social media platform. The structural model included specified demographic, body-related, and physical-activity predictors of social physique anxiety, but these outcome-stage paths did not constitute comprehensive confounder adjustment for all associations involving the mediators. Residual and unmeasured confounding therefore remains possible.

Finally, the study did not directly identify or analyse the specific fitness-role-model content encountered by participants. It therefore cannot determine whether muscularity emphasis, thinness emphasis, sexualized presentation, transformation narratives, exercise-performance displays, or health-oriented messages show different associations with upward comparison and body self-discrepancy. Nor did the study examine factors that may make students more or less likely to engage in comparison, such as body-ideal internalization, appearance investment, self-esteem, media literacy, perceived content authenticity, or existing body-image concerns. Future studies could integrate content analysis, experimental exposure, digital behavioral tracking, and longitudinal surveys to determine which combinations of content characteristics and individual susceptibility are most strongly associated with social physique anxiety.

## 5 Conclusion

This study examined the association between exposure to fitness role models on social media and social physique anxiety among college students, with upward body comparison and body self-discrepancy specified as theoretically ordered mediators. In the latent-variable model that included the specified covariate paths to social physique anxiety, greater exposure was positively associated with upward body comparison, body self-discrepancy, and social physique anxiety. Upward body comparison and body self-discrepancy each accounted for a statistically significant indirect association, and the indirect association involving both constructs was also statistically significant. Because the direct association remained significant after the mediators were included, the association between exposure and social physique anxiety was not fully accounted for by the specified indirect paths. Supplementary observed-score analyses, including the alternative dimension-level discrepancy × importance operationalization of body self-discrepancy, produced broadly similar indirect-association patterns.

The findings suggest that fitness-role-model content may be relevant not only as a source of exercise information and health-oriented motivation, but also as a social context in which bodies are evaluated. Students who reported greater exposure also reported stronger tendencies to compare their bodies with apparently superior fitness targets and greater perceived discrepancy between their actual and ideal bodies. Both forms of body evaluation were positively associated with anxiety about how one’s physique might be observed or judged by others. However, the findings do not establish that all students respond to fitness content in the same way or that such content is inherently harmful or harmless.

The study contributes by integrating externally referenced upward body comparison and internally perceived body self-discrepancy within a single model of social physique anxiety. It also conceptualizes fitness-role-model exposure as a higher-order construct comprising passive browsing and active participation, thereby distinguishing specific engagement with fitness-related content from general social media use. The findings further support treating social physique anxiety as a specific concern about the social evaluation of the physique rather than as a broad label for body dissatisfaction. Nevertheless, the measurement evidence for the exposure construct remains preliminary, and the separate associations of passive browsing and active participation require further examination.

Given the cross-sectional and self-report design, the results should be interpreted as evidence of concurrent direct and indirect associations rather than causal effects or a demonstrated temporal psychological sequence. Reverse-direction and alternative-ordering models remain plausible. Future research should use longitudinal and experimental designs, directly compare alternative models, further evaluate the measurement properties of the exposure measure in independent samples, and investigate the content characteristics and individual susceptibilities that make upward body comparison and perceived self-discrepancy more or less likely.

## Data Availability

The original contributions presented in the study are included in the article/supplementary material, further inquiries can be directed to the corresponding author.
